# Review and Analysis of Tumour Detection and Image Quality Analysis in Experimental Breast Microwave Sensing

**DOI:** 10.3390/s23115123

**Published:** 2023-05-27

**Authors:** Tyson Reimer, Stephen Pistorius

**Affiliations:** 1 Department of Physics & Astronomy, University of Manitoba, Winnipeg, MB R3T 2N2, Canada; 2 CancerCare Manitoba Research Institute, Winnipeg, MB R3E 0V9, Canada

**Keywords:** breast imaging, microwave imaging, breast microwave imaging, breast cancer, breast cancer detection

## Abstract

This review evaluates the methods used for image quality analysis and tumour detection in experimental breast microwave sensing (BMS), a developing technology being investigated for breast cancer detection. This article examines the methods used for image quality analysis and the estimated diagnostic performance of BMS for image-based and machine-learning tumour detection approaches. The majority of image analysis performed in BMS has been qualitative and existing quantitative image quality metrics aim to describe image contrast—other aspects of image quality have not been addressed. Image-based diagnostic sensitivities between 63 and 100% have been achieved in eleven trials, but only four articles have estimated the specificity of BMS. The estimates range from 20 to 65%, and do not demonstrate the clinical utility of the modality. Despite over two decades of research in BMS, significant challenges remain that limit the development of this modality as a clinical tool. The BMS community should utilize consistent image quality metric definitions and include image resolution, noise, and artifacts in their analyses. Future work should include more robust metrics, estimates of the diagnostic specificity of the modality, and machine-learning applications should be used with more diverse datasets and with robust methodologies to further enhance BMS as a viable clinical technique.

## 1. Introduction

The standard method of breast cancer detection is X-ray mammography; however, the benefits of regular breast cancer screening with mammography have been a subject of debate, in part due to the sizable false-positive rate of 20–60% (cumulative risk of a false-positive after ten mammograms) [1] and the modality’s use of ionizing X-ray radiation. Other imaging modalities currently used for breast cancer detection, including magnetic resonance imaging (MRI) and ultrasound, have low specificity, are costly and time-consuming, require a trained operator [2], and are not ideal as an independent screening tool.

Microwave sensing has been investigated as a potential breast cancer detection technique for several decades [3], and the field remains active today [2]. The modality relies on the observed contrast in the dielectric properties of malignant and healthy tissues [4,5,6] to differentiate between healthy and cancerous breast tissues. However, several remaining challenges must be addressed before the modality is ready for clinical use. A primary challenge is the lack of robust image reconstruction methods.

Reconstruction methods in microwave sensing can be broadly grouped into two families: tomographic and radar approaches. Detailed reviews of these reconstruction methods can be found in [7,8,9]. Microwave tomography aims to reconstruct a quantitative image of the complex permittivity distribution in the breast by solving the inverse electromagnetic scattering problem. Radar methods create qualitative images by propagating signals onto the spatial domain under a particular propagation assumption. This model typically assumes ray propagation of the microwave signal, a homogeneous propagation speed in the medium, and assumes that dispersion, multiple scattering, and signal attenuation within the breast tissues are negligible [7,8,9,10].

Tomographic approaches have some advantages because the reconstructions of the complex permittivity directly describe the tissue properties that govern the contrast in malignant and healthy tissues at microwave frequencies. However, these approaches also face challenges due to the ill-posed nature of the inverse scattering problem [7,8,9,10].

Radar-based image reconstruction methods have seen more widespread experimental use than tomographic methods. Several research groups have built and evaluated radar-based systems [11,12,13,14]. Most of the radar-based image reconstruction techniques use the delay-and-sum (DAS) beamformer [15] or its derivatives, including the delay-multiply-and-sum (DMAS) beamformer [16] and the improved delay-and-sum (IDAS) beamformer [17]. Other review articles have summarized and compared the individual reconstruction techniques [7,8,9,10].

The analysis of the quality of reconstructed images is an important aspect of research into any diagnostic imaging modality. The quality of reconstructed images can be evaluated quantitatively, using image quality metrics, or qualitatively [18]. Qualitative approaches typically consist of subjective, textual descriptions of the images (without referring to any quantitative image features) and are not well-suited for inter-image comparisons or image quality analysis due to their subjective nature [18]. Quantitative descriptions are advantageous because they allow for inter-image comparisons, facilitating the assessment of different image reconstruction and signal processing algorithms. Traditional concepts of image quality include image contrast, accuracy, noise, resolution, and artifacts [19]. A complete description of image quality is necessary to facilitate comparisons between imaging systems, image reconstruction methods, experimental techniques, and signal processing approaches in BMS.

In addition to quantitative and qualitative analysis at the individual image level, the overall performance of breast tumour detection can be evaluated with respect to diagnostic sensitivity and specificity. The sensitivity and specificity of a modality are two of the primary metrics of clinical relevance and must be considered in tandem when evaluating the efficacy of a diagnostic technique [18]. A sensitive method with poor specificity may not be useful as a diagnostic tool, particularly for breast cancer screening, where many more true negatives (healthy scans) exist in the screening population than true positives (scans of tumour-containing breasts). The rigorous estimation of the potential diagnostic performance of BMS is another existing challenge within the research field [18].

Notably, much of the research effort in BMS focuses on developing algorithms for data processing and image reconstruction in simulation studies. Many research groups have presented simulated results with significant progress in these areas, and the eventual translation from simulation to clinical experimental research is eagerly awaited. However, experimental work presents unique challenges that are not readily addressed in simulation, and any clinical technique must be demonstrated first by experiment. Nevertheless, significant progress has been made in experimental investigations, both in phantom-based experiments and in clinical trials [20].

While several review articles describing the state of BMS research have been published [2,7,8,9,10,20,21,22,23], including system design [7,8,20], reconstruction techniques [7,8,9,10], antenna design [21], and dielectric properties studies [7,8,9,10,20,23], little attention has been given to the aforementioned challenges—namely image quality analysis and the potential diagnostic performance of the modality.

Only one review has examined the results of tumour detection in some clinical studies [18], but did not describe all estimates of the diagnostic performance of BMS. The review by Porter and O’Loughlin in [18] described some of the challenges to demonstrating the potential clinical efficacy of the modality, namely the variation in reporting standards across studies, variation in system design across studies, and small sample sizes. This review [18] focused on summarizing the existing results, rather than critically analyzing the methods used in the research articles examining the diagnostic potential of BMS. This article was also the first review to describe image quality analysis in BMS, but a scoping review was not performed, and several existing image quality metrics were not addressed. Additionally, this article did not consider machine-learning methods in BMS.

This article presents a scoping review of the BMS literature and focuses on experimental investigations into the use of microwave sensing for breast cancer detection, specifically examining image quality analysis and existing estimates of the diagnostic potential in both image-based and machine-learning-based diagnosis. An exhaustive search of the literature resulted in 184 papers fitting the review and research criteria [11,12,13,14,17,24,25,26,27,28,29,30,31,32,33,34,35,36,37,38,39,40,41,42,43,44,45,46,47,48,49,50,51,52,53,54,55,56,57,58,59,60,61,62,63,64,65,66,67,68,69,70,71,72,73,74,75,76,77,78,79,80,81,82,83,84,85,86,87,88,89,90,91,92,93,94,95,96,97,98,99,100,101,102,103,104,105,106,107,108,109,110,111,112,113,114,115,116,117,118,119,120,121,122,123,124,125,126,127,128,129,130,131,132,133,134,135,136,137,138,139,140,141,142,143,144,145,146,147,148,149,150,151,152,153,154,155,156,157,158,159,160,161,162,163,164,165,166,167,168,169,170,171,172,173,174,175,176,177,178,179,180,181,182,183,184,185,186,187,188,189,190,191,192,193,194,195,196,197,198,199,200,201,202]. This article reviews and critically analyzes the methods used for image quality analysis and discusses how current methods relate to traditional aspects of image quality, including image resolution, noise, contrast, accuracy, and artifacts. This review also critically analyzes the estimated diagnostic performance of microwave-based breast cancer detection in both image-based and algorithmic (machine learning) detection approaches.

## 2. Review Methodology

A scoping review of the breast microwave sensing literature was performed to identify all published work that experimentally investigated tumour detection or image quality analysis. Articles that exclusively presented results using simulated data were excluded from this review, as were articles that:

- Used non-physical phantom materials (e.g., metal as a tumour analog);
- Examined contrast-enhanced breast microwave sensing;
- Examined multimodality imaging (e.g., using MRI-based prior information for microwave image reconstruction).

These exclusion criteria were used to limit the scope of the review to experimental tumour detection and image quality analysis in breast microwave sensing. Additionally, all conference papers that were expanded into journal articles were excluded, and only the journal articles were included in the review to prevent the double-counting of papers.

**Figure 1:**
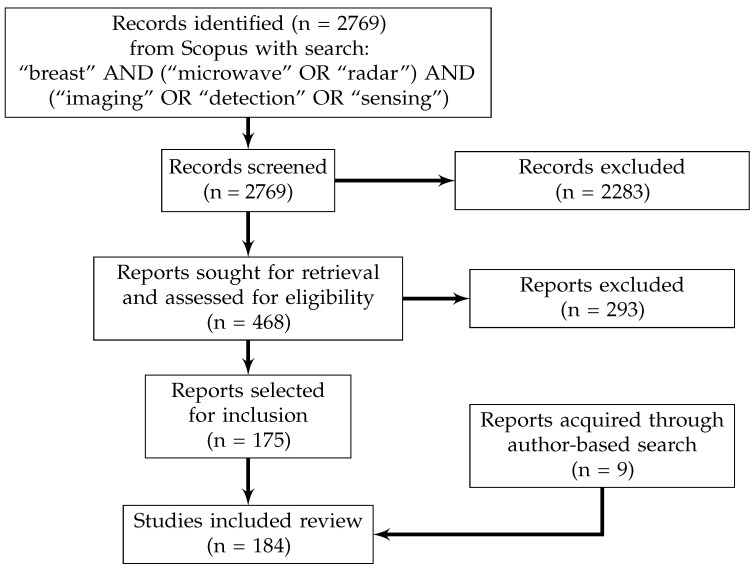
Flowchart describing review methodology. In total, 184 articles were identified for inclusion after reviewing 2769 search results from Scopus.

The Scopus search engine was used to identify all papers published before February 17th 2023 that satisfied these criteria. The search was performed with the following keywords: “breast” AND (“microwave” OR “radar”) AND (“imaging” OR “detection” OR “sensing”), and the search results were limited to journal articles and conference papers. All studies in the search results (n = 2769) were initially evaluated for inclusion based on their title and abstract. The studies that were selected for possible inclusion based on their abstract and title (n = 468) were then evaluated with respect to the inclusion and exclusion criteria. One hundred seventy-five papers were identified for inclusion in this review [11,12,13,14,24,25,26,27,28,29,30,31,33,34,35,36,37,38,39,40,41,42,43,44,45,46,47,48,49,51,52,53,54,55,56,57,58,59,60,61,62,63,64,65,66,68,69,70,71,73,74,75,76,77,78,79,80,81,82,83,84,85,86,87,88,89,90,91,92,93,94,95,96,97,98,99,100,101,102,103,104,105,106,107,108,109,110,111,112,113,114,115,116,117,118,119,120,121,122,123,124,125,126,127,128,129,130,131,132,133,134,135,136,137,138,139,140,141,142,143,144,145,146,147,148,149,150,151,152,153,154,155,156,157,158,159,160,161,162,163,164,165,166,167,168,169,170,171,172,173,174,175,176,177,178,179,181,182,183,184,185,186,187,188,189,190,191,192,195,196,197,198,199,200,201]. Nine additional papers [17,32,50,67,72,180,193,194,202] that were not in the Scopus search results but which nonetheless fit the inclusion criteria were also selected for inclusion in this review. Therefore, there were 184 papers selected for inclusion [11,12,13,14,17,24,25,26,27,28,29,30,31,32,33,34,35,36,37,38,39,40,41,42,43,44,45,46,47,48,49,50,51,52,53,54,55,56,57,58,59,60,61,62,63,64,65,66,67,68,69,70,71,72,73,74,75,76,77,78,79,80,81,82,83,84,85,86,87,88,89,90,91,92,93,94,95,96,97,98,99,100,101,102,103,104,105,106,107,108,109,110,111,112,113,114,115,116,117,118,119,120,121,122,123,124,125,126,127,128,129,130,131,132,133,134,135,136,137,138,139,140,141,142,143,144,145,146,147,148,149,150,151,152,153,154,155,156,157,158,159,160,161,162,163,164,165,166,167,168,169,170,171,172,173,174,175,176,177,178,179,180,181,182,183,184,185,186,187,188,189,190,191,192,193,194,195,196,197,198,199,200,201,202]. Figure 1 displays this review methodology.

## 3. Image Quality Analysis

Image-based analyses have been the primary form of tumour detection in the BMS literature—of the 184 papers identified in this review, 164 (89%) exclusively examined image-based tumour detection. However, image-based analysis has been primarily qualitative. Of the 164 papers that utilized image-based tumour detection, 89 (54%) presented images without any quantitative analysis of the image quality (see Table A1 in the Appendix A). In all cases where quantitative image quality analysis was performed, the quantitative techniques relied on single pixel/voxel responses or *a priori* knowledge of the actual tissue dielectric properties or geometries. Eleven unique image quality metrics have been defined in the literature, seven of which aim to describe image contrast. Table 1 and the following discussion describe these metrics:

- The signal-to-clutter ratio (SCR);
- The signal-to-mean ratio (SMR);
- The mean-to-mean ratio (MMR);
- The tumour-to-fibroglandular response ratio (TFRR);
- The contrast-to-clutter ratio (CCR);
- The clutter-to-tumour ratio at threshold *t* (C/T${}_{t}$);
- The localization error (LE);
- The mean squared error (and the associated family of error metrics) (MSE);
- The full-width at half-maximum area (FWHM);
- The $f_{1}$ and *f* metrics presented in [70];
- The structural similarity index measure (SSIM).

Several quality metrics have been proposed to describe image quality, including the SCR, SMR, MMR, TFRR, CCR, and C/T${}_{t}$. The SCR has been the most commonly used metric in breast microwave imaging and was used in 22 of the 76 papers which presented quantitative image analysis as displayed in Table 1, but these metrics share a common mathematical structure. The definitions of these metrics vary, as shown in Table 1, but they all attempt to measure the contrast of the image. None of these metrics are robust due to their reliance on single-pixel intensities (e.g., the maximum pixel intensity is used in the SCR, SMR, TFRR, and CCR) or their dependence on the definition of the imaging domain or breast size and density (as in the SMR and MMR). These metrics also rely on defining a tumour region and a non-tumour region, which typically requires a priori knowledge of the tumour location within the image, making these metrics unsuitable for use outside of controlled experimental conditions where the tumour location can be measured accurately and precisely.

The SMR and MMR metrics are dependent on the choice of imaging domain (due to the very low-intensity responses that occur outside of the breast tissues in the coupling medium or air), breast density (due to the higher dielectric properties of fibroglandular tissue than adipose tissue), and breast size. An independent variation in any of these three parameters may change the value of the mean intensity in the clutter region, making these techniques unsuitable for diagnosis without additional corrections. For example, a small, dense breast may have a smaller SMR/MMR than a large, low-density breast with a single fibroglandular inclusion located relatively superficially. In addition to these dependencies on factors unrelated to tumour presence (breast size, imaging domain size), the authors of [132] also identified that the metric fails in the case of a large lesion. In describing an individual patient with a 4 cm carcinoma, the researchers stated, “The extremely large size of such carcinoma leads to a low Max/Avg” [132], indicating that the interpretation of this metric must account for other factors, including the lesion size, which may not be known a priori.

The terms in Table 1 are the most commonly used terms for each of these mathematical definitions, but significant confusion exists in the BMS literature surrounding the terms and mathematical definitions of these metrics. For example, while the definition in Table 1 is the most common in the literature, several other definitions have been used. The SCR was defined in [142,145] as the ratio between the maximum pixel response in an image acquired from a tumour-containing phantom to the intensity in the corresponding pixel in an image acquired from a tumour-free phantom. This definition was referred to as the “S/C ratio” in [198]. The “S/C ratio” was also used in [131,132], but in these studies, it referred to the ratio of the maximum pixel intensity to the average pixel intensity. The SCR has also been defined in [11] as the ratio of the mean pixel intensity within a target region of interest (ROI) to the mean pixel intensity outside the ROI. The ratio of the square of the maximum tumour response to the standard deviation of the background pixel intensities has also been defined as the SCR [26,69]. The term SCR has also been used as an image quality metric but was not defined within the manuscripts where it was used [68,98]. The definition of the SCR in Table 1 was used to define the tumour-to-clutter ratio (TCR) [24]. A metric similar to the SCR was defined in [58] as the ratio of peak clutter energy to peak tumour energy, using the definition of the SCR, except the inverse of the argument of the logarithm is used [58]. The term “signal-to-max ratio (SMXR)” was used in [78,142] but was defined as the SCR is defined in Table 1.

This is also true for the SMR, which has been described by other names (e.g., “MAX/AVG” in [132,133,162], “peak/mean” in [182], and the tumour-to-mean ratio in [24], and “S/C” in [131]), and has been given other mathematical definitions (e.g., [178,190]). The MMR has also been used under different names [14,99,164,173,178,190]. Other work has described an SMR without definition [56,68,98,99,153,154].

The CCR is unique because of its use of the standard deviation of image intensities, σ, as described in Table 1. However, the inclusion of this variable in the denominator obfuscates the interpretation of the CCR as a description of image contrast—a relatively noisy image with relatively high contrast may have the same CCR as a relatively low-noise image with relatively low contrast.

The LE, MSE, and SSIM metrics aim to describe image accuracy, but are also non-robust. The LE describes a single specific aspect of image accuracy: the accuracy of the target (typically a tumour) positioning in the reconstructed image. This metric is non-robust due to its reliance on a single pixel intensity to identify the tumour response in the image. The MSE (and similar metrics that sum over all pixels in the image, including the mean absolute error, the total squared error, residual error, etc.) provides a summary measure of the image accuracy, but is only applicable when the underlying object properties are well known. This limits the applicability of this metric in patient studies where the underlying microwave properties of the tissues are not well known; while image registration using traditional image modalities, including MRI and CT, may aid in this circumstance, image registration is a challenging task and the MRI/CT reconstructions do not necessarily map to the underlying microwave properties. Additionally, these metrics average over the entire image and do not provide insight into reconstruction accuracy at the level of individual image features (e.g., accurate reconstruction of a particular tissue or region of tissue). The SSIM is an additional summary measure of image quality; it is a measure of the similarity of two images, first proposed by [203], and is commonly used in medical imaging. The application of the SSIM in BMS [176] has the same limitations as the MSE. It is a summary image accuracy metric, and requires complete a priori knowledge of the ground truth property distribution.

The area of the full-width at half-maximum (FWHM) of the image intensities has also been used as an image quality metric [101,114,189,190], but this metric does not clearly describe the quality of the image. In an image with a clear tumour response, the FWHM may be expected to be approximately the same size as the tumour, but this may also be true in an image of a relatively homogeneous breast with a single relatively small fibroglandular inclusion. The presence of fibroglandular tissue may also produce responses that are included in the FWHM of the image, given that the contrast in the dielectric properties of malignant and fibroglandular tissue may be as small as 1.1:1 [5,6].

Unique metrics were proposed by the authors of [70]. The authors introduced the $f_{1}$ image quality metric,

(1) $$f_{1}=\frac{I_{\max}}{N\cdot A}$$

where *A* is the area of the FWHM of the image and *N* is the number of distinct areas within the FWHM. A modified version of this metric was also defined in [70] with the form $f=f_{1}P$ where

(2) $$P=\min\left({\left(\frac{d}{3\;\mathrm{mm}}\right)}^{3},\;\;1\right)$$

and *d* is the distance from the boundary of the imaging domain to the pixel with intensity $I_{\max}$. This modifying factor *P* was introduced due to the observed presence of “small and very bright artifacts [that] appear at the edges of the image” [70]. The penalty distance of 3 mm was selected due to an average skin thickness of 2 mm and a 1 mm “buffer” [70].

These metrics were developed for permittivity estimation [70] but may be considered a measure of image quality. A large value of the metric indicates a high-quality image, signifying an image with a high-intensity maximum and a small FWHM area. This metric has demonstrated utility in determining the optimal permittivity estimate for image reconstructions from a single scan [70] but is not appropriate for inter-image comparisons. Both the $f_{1}$ and *f* metrics are proportional to the maximum image intensity and are therefore not appropriate for inter-image comparisons in radar-based imaging due to the qualitative nature of radar reconstructions. The metric has not been applied to quantitative tomographic reconstructions but is also not useful for inter-image comparisons due to the inter-patient variations in dielectric properties. Measurements of the dielectric properties of malignant and benign breast tissues demonstrate significant overlap in the malignant and benign properties of tissues between patients [5].

The image quality metrics described in this section are summarized in Table 1. The $f_{1}$ and *f* metrics [70] were excluded from Table 1 due to their limitations for inter-image comparisons.

Notably, all existing image quality metrics aim to describe image contrast or accuracy. A complete description of image quality includes descriptions of the spatial resolution, contrast, contrast resolution, noise, accuracy, and artifacts.

**Figure 2:**
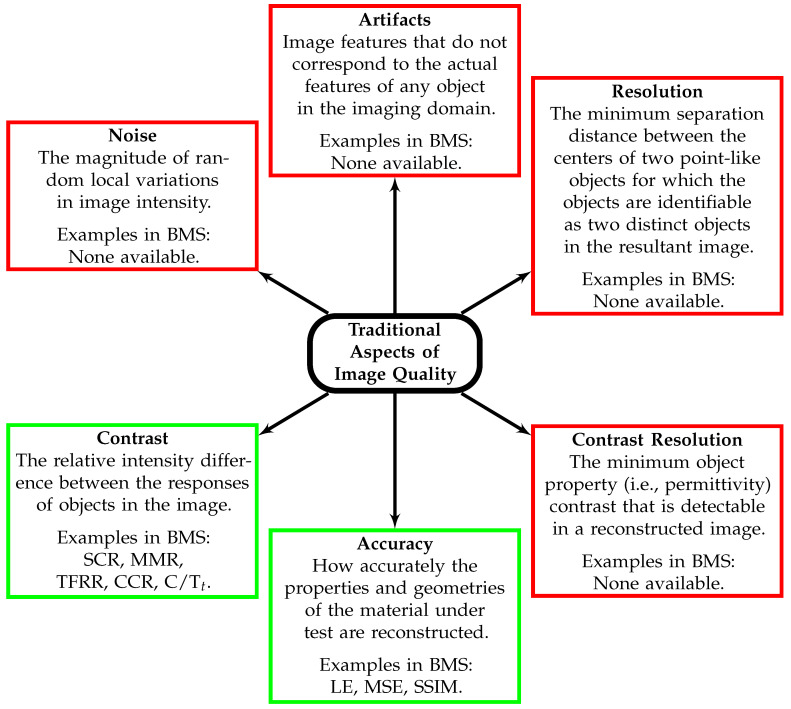
Traditional aspects of medical image quality [19] and existing image quality metrics in breast microwave imaging. Aspects described in boxes with red borders have not been addressed in the BMS literature; aspects in boxes with green borders have been addressed in the BMS literature.

Figure 2 describes these traditional aspects of image quality and illustrates the focus of existing BMS research. Each of these aspects describes an important component of image quality, and each must be considered when evaluating the reconstruction quality of a particular system or method. The narrow focus on contrast in the existing BMS literature limits the ability to compare the quality of images across the multiple important dimensions of image quality.

In particular, the presence of artifacts in reconstructed images has not been well addressed in the research literature. Artifacts are image features that do not correspond to the physical reality of the interrogated object (e.g., a high-intensity region within the image that does not correspond to an actual high-scattering tissue component within the breast). In radar-based image reconstruction, a common artifact observed in reconstructions is the presence of a localized high-intensity region that does not correspond to the known location of a tumour. This hotspot artifact is readily discerned in the reconstructed images of healthy breasts in several articles, including figures in [67,100,110,129,190].

**Figure 3:**
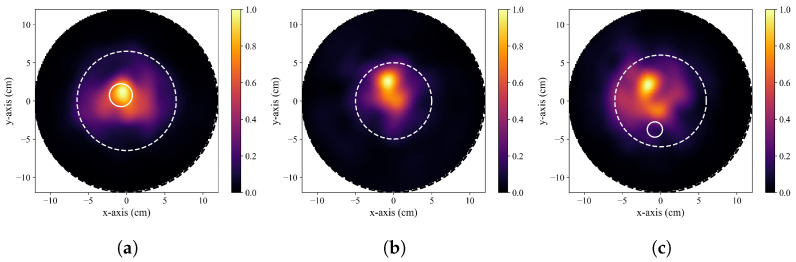
Coronal DAS reconstructions of breast phantoms from the open-access dataset presented in [118]. The dashed white lines indicate the approximate phantom boundary, the black dashed lines indicate the antenna trajectory during the scan, and the solid white circles in (**a**,**c**) indicate the known tumour positions. (**a**) Displays a reconstruction with a tumour response that corresponds to the known tumour position, (**b**) displays a reconstruction of a phantom that did not contain a tumour but has a prominent tumour-like response (a false-positive), and (**c**) displays a reconstruction of a tumour-containing phantom that has a prominent tumour-like response that does not correspond to the known tumour position (a false-positive).

To illustrate this artifact, typical DAS reconstructions are displayed in Figure 3, where only Figure 3a accurately displays the true tumour response. In Figure 3a, a high-intensity localized region is observed within the known tumour location. In Figure 3b, a similar hotspot is observed, but this is an artifact in this reconstruction of a healthy phantom (no tumour inclusion). Figure 3c displays a reconstructed image of a tumour-containing phantom with a clear tumour-like response that does not correspond to the actual tumour position within the phantom. The images reconstructed in Figure 3 were produced by our research team using publicly available data (dataset described in [118]).

The presence of this hotspot artifact should be described in studies that utilize image-based tumour detection in BMS, and a more thorough examination of its impact on the potential specificity of the modality should be presented in future work within the research field. Only 52 (32%) papers that presented image-based analyses did so using images of healthy patients or phantoms (i.e., no tumour was present) (see Table A1(ii)). Comparisons between healthy and unhealthy images are necessary to identify potential artifacts like the hotspot artifact and to determine the diagnostic performance of image-based tumour detection. Additionally, only 25 articles performed quantitative comparisons of healthy and unhealthy images (see Table A1(iii)). Future work in image-based tumour detection should present analyses using both healthy and unhealthy phantoms and patients.

## 4. Estimates of the Diagnostic Potential of Image-based Tumour Detection

Several research groups have attempted to estimate the diagnostic performance of image-based tumour detection and twelve articles have reported estimates of the diagnostic performance of image-based BMS (see Table A1(iv)). This section focuses on these key articles that have pushed to estimate the diagnostic performance of BMS. Eleven of these articles provided sensitivity estimates, ranging from 63% [47] to 100% [141,170], but only four articles have estimated the specificity, and specificity estimates range from 20% [100] to 65% [133]. Table 2 describes the diagnostic estimates presented in each of these articles.

The majority of estimates have come from patient datasets (see Table A1(v)) with sample sizes varying from 5 patients in [141] up to 225 patients in [143]. Two articles [100,120] estimated the diagnostic performance using breast phantoms, under well-controlled experimental conditions. Notably, the poorest specificity estimates in the literature are found in these works (20% in [100] and as low as 40% in [120]); the majority of trials performed using patient data were not designed to evaluate specificity. The works in [47,94,111,141,143,170] all exclusively examined patients with known breast lesions.

Various diagnostic criteria have been used in these works. The phantom-based work in [100,120] used a quantitative tumour-detection criteria that compared the SCR of each image to a threshold of 1.5 dB—if the SCR was above this threshold, the image was annotated as containing a tumour response. Images were then labelled as true positives by comparing the localization error to the known tumour radius. In [100], an exception was applied to account for a priori knowledge about the presence of hotspot artifacts near the skin of the phantoms so that reconstructions exhibiting this artifact were not considered false positives. Notably, of the images (incorrectly) annotated as tumour-free, 94% received this annotation due to the exception (i.e., the high-intensity response was within 10 mm of the boundary of the imaging domain, which was defined by the breast boundary), and only 6% of the reconstructions that were labelled as tumour-free did not meet the SCR criterion [100]. This indicates that while the reconstructions consistently displayed a localized high-intensity region (as indicated by the proportion which met the SCR criterion for tumour detection), localization was poor, and this high-intensity region was often not due to the tumour itself. While this work estimated the diagnostic sensitivity and specificity, the tumour-free dataset was too small (n = 5) to draw significant conclusions regarding the specificity of the method, and the subjective selection of SCR threshold (1.5 dB) does not provide a full description of the potential diagnostic performance of the method. Notably, the work of Reimer et al. in [120] is the only published research comparing the diagnostic performance of multiple image reconstruction methods (see Table 2). The authors also presented an open-source analysis using an open-access dataset [120]. This was the only article in the literature that reported the diagnostic performance using open-source analysis and an open-access dataset.

While phantom-based investigations lend themselves to quantitative diagnostic criteria due to the well-controlled experimental conditions (where tissue properties and geometries are known a priori), diagnosis in patient-based trials is more challenging. The work in [93,132,133,134,201] applied quantitative tumour detection measures to evaluate the diagnostic performance. The authors of [132,133,134] used the “MAX/AVG” metric to diagnose the presence of a tumour. A threshold value of this metric was determined from the image dataset in these articles, and the reported sensitivities describe the percentage of images corresponding to tumour-containing breasts that had metric values greater than this threshold. However, the MAX/AVG metric may not be suitable as a universal metric for tumour detection due to its dependence on the entire imaging domain—the MAX/AVG metric is directly influenced by breast size. A larger tumour-containing breast is expected to have a smaller MAX/AVG value than a smaller breast containing the same tumour and healthy tissue components, because the larger volume of healthy tissue responses in the large breast will skew the average image intensity to a smaller value. Additionally, there is limited utility in extending a sample-scale threshold value to a patient population.

Region-of-interest (ROI)-based analyses, where the intensities in an ROI were compared to intensities outside the ROI or to intensities in other images within the same ROI, were used in [94,201]. The work in [201] is notable as being the first published work to estimate the diagnostic performance of BMS and the only existing article that estimated the diagnostic performance based on tomographic image reconstruction. The authors of [201] used a non-blind approach to tumour detection where the mean permittivity and conductivity values in an ROI (obtained from conventional imaging modalities, i.e., obtained using a priori information of the tumour position) were compared in images of tumour-containing breasts to images of healthy breasts. The ROIs used for the reconstructions of healthy breasts were drawn using the previously drawn ROI for a tumour-containing reconstruction. This approach ensured that the ROIs in the healthy breasts were dependent on the ordering of the dataset, but no investigation into this effect was performed. The work in [94] did not rely on a priori information, and instead created the ROI through image thresholding. The ROI was labelled as corresponding to a lesion based on its size, solidity, and contrast [94]. Notably, four of the twenty-four images in [94] contained a response that was satisfied the tumour-detection criteria, but did not have an accurate position (i.e., the tumour-like response did not occur at the known tumour location). The authors did not discuss whether these responses were rightfully attributed to the breast lesion or were an artifact, and the magnitude of the localization error was not described. These lesion-like responses may not be attributable to the breast lesion.

Half of the research in image-based patient diagnosis has been performed using subjective diagnostic criteria [49,111,141,143,170]. These articles relied on subjective judgments of diagnosticians to determine the presence of a cancerous response in the reconstructed image. These studies were also non-blind, and the diagnosticians may have known a priori that the images corresponded to breasts with known abnormalities—this is explicit in [170], where the reviewing radiologist was “told only that the patient had breast cancer” [170]. No current work has implemented single- or double-blind protocols to estimate the diagnostic performance of BMS. This limits the impact of the reported diagnostic estimates, and is particularly limiting when subjective diagnosis by a human reviewer was performed with the a priori knowledge of a known breast abnormality.

**Figure 4:**
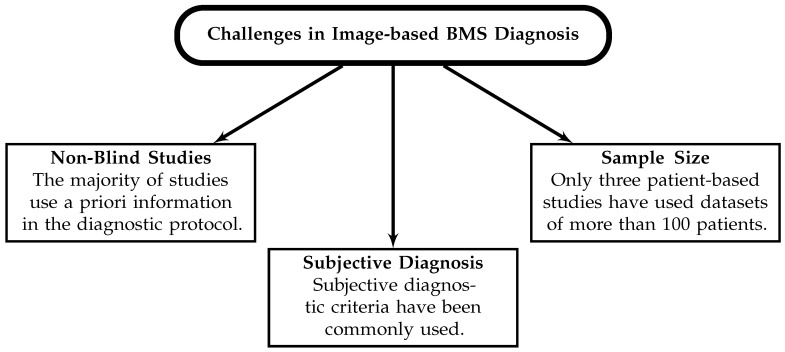
Current challenges in image-based estimation of the diagnostic performance of BMS. Non-blind studies [100,111,120,141,143,170,201], subjective diagnosis [49,111,141,143,170], and sample size limitations were common [48,143,201].

Despite the existing efforts to estimate the sensitivity of BMS, the impact and validity of existing estimates is limited due to small sample sizes, subjective diagnostic processes, the use of a priori knowledge in objective diagnostic processes, and the lack of single- or double-blind protocols. Figure 4 summarizes these challenges. Subjective diagnostic criteria limit reproducibility and obfuscate comparisons between studies. There has been a particular focus on sensitivity in BMS, but the sensitivity and specificity of a modality must be considered in tandem when evaluating the efficacy of a diagnostic technique. A sensitive method with poor specificity may not be useful as a diagnostic tool, particularly for breast cancer screening, where many more true negatives (healthy scans) exist in the screening population than true positives (scans of tumour-containing breasts). Obtaining a sufficiently large sample size for statistical considerations in a clinical trial is also challenging, but blind phantom studies could be performed readily to address this. Future work may consider performing blind phantom-based trials to demonstrate the efficacy of the modality. Phantoms offer several utilitarian advantages over patient data, and while patient-based trials are ultimately the gold standard, little investigative work has been done to evaluate the diagnostic performance of BMS with phantoms before clinical trials were performed. Only two articles have attempted to estimate the diagnostic performance using phantom datasets [100,120], published in 2019 and 2021, while several patient trials have been ongoing since 2007 [201]. Robust demonstration of the diagnostic performance of the methods in BMS should be achieved under the controlled experimental conditions offered by phantoms before further, larger clinical trials are performed.

## 5. Machine Learning-Based Tumour Detection

### 5.1. Appropriate Machine Learning Methodology

The recent advancements in machine-learning (ML) algorithms have increased interest in automated and objective diagnosis throughout medical imaging, and these techniques have been applied to both simulated [204,205] and experimental data [112,118] in breast microwave sensing. ML methods for medical imaging techniques have not been as widely investigated as image-based tumour detection methods, but the results that have been presented are promising. ML techniques do, however, face unique challenges. ML techniques can be extremely powerful tools, capable of addressing numerous problems in a wide range of problem domains. Like any tool, these methods can, however, be misused. Appropriate ML methodology must be used in any application of ML methods to ensure the results obtained with an ML model are responsibly reported and reasonably approximate the real-world performance of the model. Specifically, the dataset size, dataset diversity, and training/testing methods must be carefully considered when evaluating the application of ML methods to a particular problem. Unfortunately, basic ML methodology is rarely followed in the BMS literature.

ML methods, generally, require a large dataset (relative to those present in the BMS literature to date) for model training and evaluation. Most work has investigated the application of ML methods on no more than a few hundred individual samples (phantom scans) [76,82,104,112,118,138]. Two articles have used datasets of more than 1000 samples [79,119], but the dataset size is not the only dataset parameter that must be considered. Data diversity, the variation between individual samples in the dataset, is also essential to ensure that, during training, the ML model is exposed to data that represents the real-world data landscape. Insufficient data diversity will likely limit performance when the model is exposed to novel data (e.g., training using data from only relatively low-density breasts will likely result in poor performance on relatively dense breasts). When evaluating or testing the trained ML model, diversity is also important. The testing set should consist of new or unseen data—data as dissimilar to the training samples as individual samples in the real-world would be from each other. In BMS, the training and testing samples should be as dissimilar as the data collected from breast scans obtained from different people. If the diversity of the dataset is limited so that samples in the training and testing sets are more similar than data obtained from different people (in the real-world deployment scenario), then estimates of the model performance on the testing set will be overly optimistic. Without a sufficiently large dataset, model training and evaluation are challenging, and without sufficient dataset diversity, the target population (on which the trained model would be deployed) may not be wholly represented, limiting the real-world potential of the model.

**Figure 5:**
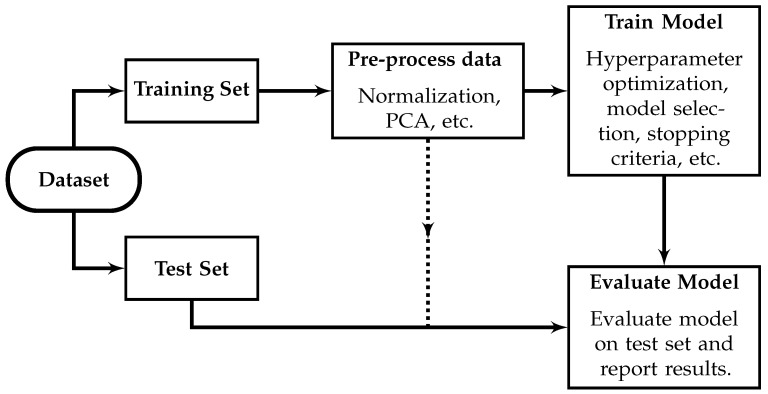
Fundamentals of appropriate ML methodology. Dataset is split into training and testing subsets before any further analysis. The training set is exclusively used to learn any pre-processing transformation, for hyperparameter optimization, model selection, and stopping criteria selection. The test set is only used at the model evaluation stage. The dashed line indicates that transformations learned during pre-processing (e.g., PCA) may be applied to the test set before model evaluation, but must not be obtained using information from the test set.

A robust training and testing methodology is essential to appropriately interpret the results obtained with an ML model. A separate, unique, unseen test set is necessary to evaluate the performance of a trained ML model, and no information from the test set should influence the trained ML model. To prevent data leakage, data from the testing set should not be used for pre-processing (e.g., Z-score normalization, principal component analysis, etc.), hyperparameter optimization, or training [206,207]. The most extreme case of data leakage involves using the same samples for training and testing. However, even the use of test set performance metrics to influence the choice of ML model or the choice of ML model architecture qualifies as data leakage and can result in overly optimistic estimates of the model performance [206]. The effect of data leakage can be significant and results obtained when data leakage has occurred must be carefully interpreted and may not be an accurate estimate of the model performance. Figure 5 illustrates appropriate ML methodology.

### 5.2. Analysis of Existing Estimates of the Diagnostic Performance of Machine Learning in Breast Microwave Sensing

This section discusses the key works that have estimated the diagnostic performance of ML in BMS; while several articles have attempted to use ML methods in BMS, the use of ML tools has not been rigorous, and methodological errors are common. Additionally, the generalization of trained ML models in BMS is questionable. No trained model has been evaluated using data obtained from a different imaging system or at a different geographical location, and published work has limited dataset diversity. Machine-learning methods have also been applied to classify phantom measurements known to have a benign or malignant tumour as being benign or malignant [205,208,209,210]. These applications do not aim to detect a tumour’s presence but rather to confirm the malignancy of a known tumour in a phantom and were, therefore, not included in this review. These studies are mentioned here for completeness as there are relatively few applications of machine learning for tumour detection within breast microwave sensing. Other studies, which classified known lesions in patient data [48,49,93,196], were included due to their use of patient data.

There have been fifteen studies that have estimated the diagnostic performance of microwave-based sensing for algorithmic breast cancer detection (see Table A1(vi)). The techniques used in these studies include logistic regression, support vector machines (SVM), linear discriminant analysis (LDA), quadratic discriminant analysis (QDA), classifier ensembles, multilayer perceptrons (MLP), convolutional neural networks (CNN), dense neural networks (DNN), gradient boosting methods, and k-nearest neighbour classifiers (kNN). Table 3 summarizes these investigations into ML-based BMS cancer diagnosis. Three additional articles are noteworthy [80,104,113], but are excluded from Table 3. The work in [104] used the same dataset as [118] and used tree-based models, but none of the models improved upon the results in [118]. The methods and results in [113] are expanded upon in [202], and therefore only [202] is included in Table 3. The authors of [80] used a dataset that combined experimental and simulated data. This article has been excluded because of the use of simulated data in an unspecified subset of the dataset.

ML-based diagnostic methods in BMS have generally achieved better diagnostic measures than image-based approaches with larger datasets. The largest dataset used in an ML-based investigation was in [119] which used data from 1008 phantom scans. ML methods have been applied to patient (see Table A1(vii)) and phantom datasets (see Table A1(viii)). Estimates of the diagnostic performance obtained with patient and phantom datasets are similar (see Table 3).

Notably, the MammoWave system [135] has been used in five ML-based investigations [112,113,135,188,202]. These works provide the most optimistic estimates of the diagnostic performance of BMS in the literature, but the reported results are likely overestimates due to methodological limitations in these works. These works suffered from data leakage, incomplete methodological descriptions (insufficient to facilitate replication), and lacked true test sets. The work in [112] ensured that hyperparameter tuning was specific to the dataset used in the work due to the lack of a test set, as did [113,202]. Data leakage was possible or explicitly described in each of these articles; while the reported results are positive, these methodological concerns imply that the reported diagnostic estimates are likely overestimates of the true performance.

Data leakage is a common issue in ML-based investigations in BMS. Data leakage possibly occurred in five articles (see Table A1(ix)) and explicitly occurred in five articles (see Table A1(x)). Data leakage is an avoidable methodological issue that ensures estimates of the ML model performance are overestimates. Data leakage issues observed in BMS included pre-processing methods applied before train/test set splitting was performed (including PCA and Z-score normalization) and incorporating testing data into the training procedure. Several articles did not use an explicit test set. The lack of an appropriate test set limits the impact of the ML results, and while an appropriate test set requires a sufficiently large dataset, k-fold validation may be performed, where the dataset is segmented into k-subsets. For each of the subsets, the remaining dataset is used for all pre-processing, model training, and hyperparameter optimization. This process allows a reasonable estimate of the diagnostic performance. Several of these articles have also provided limited descriptions of the ML methodology that either prohibit replication and leave open the possibility of methodological issues (primarily data leakage). However, some work has been done using open-access datasets [53,104,118,119] and open-source methods [53,118,119].

Dataset diversity has been limited in BMS investigations, particularly in phantom-based studies. Breast phantoms are useful experimental tools, particularly for machine learning, where large datasets can be generated [118], but multiple measurements of the same phantom have been observed to be more similar than measurements of different phantoms [119], and repeated measurements of a given phantom may not be appropriately considered as truly unique measurements [119]. Several articles used datasets with multiple measurements of a given phantom (see Table A1(xi)). Additionally, the work in [196] treated multiple images generated from a scan of a given patient’s breast as unique samples. Notably, the authors of [119] used a leave-one-out testing strategy. The dataset used in this work consisted of 1008 phantom scans of 66 unique phantoms. The phantoms were each comprised of an adipose and a fibroglandular component (and, in some cases, a tumour). The adipose component determined the outer shape of the phantom, and, therefore, the most dominant reflections (due to the in-air imaging system). The authors trained the classifiers using data from an all-but-one adipose component, and then tested on the phantoms made with this left-out component. This procedure maximized data diversity between the training and testing sets and ensured that the trained models were tested on truly unseen phantoms. The average ROC AUC was found to be (78 ± 3)% across all phantoms, but if the testing set was constrained to consist of phantoms with breast volumes within the bounds of the training set and to only contain data from scans where the tumour was at the same vertical height of the antenna, the ROC AUC was found to be (90 ± 3)%. This article was the first to explore the effects of a limited dataset in terms of breast diversity (specifically, breast size and shape) on the diagnostic performance of a tumour detection method. The authors observed that multiple measurements of a phantom (of a given adipose component) were more similar than multiple measurements of unique phantoms. This observation implies that data diversity may significantly affect model performance.

## 6. Achievements, Challenges, and Recommendations

### 6.1. Achievements in Breast Microwave Sensing

Several research groups have developed microwave sensing systems capable of clinical operation and performed studies involving patients or volunteers [11,12,67,84,109,111,131]. Within the 184 papers examined in this literature review, experimental results of tumour detection with microwave sensing systems were presented by more than two dozen unique research groups, including multiple private corporations, with the majority of the results being reported in the last decade. The relatively large number of independent research groups to develop and evaluate custom-made microwave sensing systems is an achievement for the international BMS research community.

Microwave sensing systems are sensitive to the presence of tumours within the breast and are capable of detecting the presence of sub-centimeter lesions. The smallest reported detected lesion in a phantom had a largest dimension of 3 mm [43,199] and the smallest reported detected lesion in a patient had a largest dimension of 4 mm [48], and numerous investigations demonstrated detection of sub-centimeter tumours (see Table A1(xii)). Sensitivity estimates obtained using phantom and patient datasets with image-based diagnostic criteria range from 63 to 100% (see Table A1(iv)). System bandwidth, antenna design, and data collection protocols vary within the literature, but all current systems have demonstrated sensitivity to the tumour response.

ML methods have been more frequently used for tumour detection than image-based methods (see Table A1(v)). These methods illustrate a potential future approach whereby breast cancer diagnosis is automatically performed using low-cost microwave systems using objective and quantitative ML models. These methods are particularly well-suited to low- and middle-income countries and rural locations, where access to healthcare may be limited due to the automated diagnostic process. No trained diagnostician or reviewer may be required. This research path may be fruitful in the future.

### 6.2. Challenges in Breast Microwave Sensing

This review reveals three significant challenges face the BMS research community: the poor estimates of the modality’s specificity; the limited and poor methods of image quality analysis; and the lack of robust methods and dataset diversity in machine-learning-based investigations.

Only four articles have estimated the image-based specificity of the modality, and the existing estimates range between 20 and 65% [100,120,133,134]. These estimates do not demonstrate that microwave sensing is ready for clinical application; while sensitivity estimates range from 63 to 100%, the sensitivity of a diagnostic technique is only informative in the context of the specificity of the technique. Despite a push to perform larger clinical trials, with the largest featuring 225 patients [143], existing specificity estimates are poor. Only two articles have attempted to evaluate the diagnostic performance of the modality using phantoms under controlled experimental conditions [100,120], and the specificity estimates of 20% [100] and 56% [120] are not promising. Viable specificity of this modality must be demonstrated under experimental conditions before further patient studies should be performed, given the ethical considerations involved in clinical trials.

Image quality analysis in BMS has been limited to analyses of the image contrast and localization error. Various terms have been used for the same mathematical definitions of image quality metrics (e.g., SCR, SMR sometimes refer to the same mathematical definition), and various mathematical definitions have been applied to the same term (e.g., SCR has had multiple definitions). This lack of consistency in the definitions of image quality metrics obfuscates the analyses presented in the literature and limits the comparison of image reconstruction techniques across publications. The research community in BMS should aim to build upon the work of others and utilize consistent definitions of image quality metrics. Current image quality metrics are also limited in their usefulness due to their reliance on a priori knowledge of tissue properties and/or geometries. This requirement limits these metrics to applications in well-controlled experiments, where this a priori information is known. Most current metrics (including the most often used SCR, SMR, and localization error) rely on single pixel/voxel intensities and are therefore non-robust. Several aspects of traditional image quality have been neglected in the literature, including image resolution, noise, and artifacts. The hotspot tumour-like artifact present in reconstructions of healthy breasts is an example of this; while the artifact appears in several publications, no formal discussion or characterization of it has been presented in the literature. Future work in image-based BMS should aim to describe image quality more thoroughly.

Only one reliable estimate of the diagnostic performance obtained with ML has been presented, and while the estimate of an ROC AUC of (90 ± 3)% is positive [119], further work using more diverse datasets is necessary. The majority of existing ML-based work in BMS has not adhered to appropriate ML methodological standards. The reported estimates of the diagnostic performance of ML models in the literature are therefore overestimates.

### 6.3. Recommendations

Despite the significant progress in BMS research, several challenges remain. Several of the identified challenges must be addressed before microwave-based systems can be considered for clinical use as breast cancer detection systems. Therefore, we make the following recommendations for future work in experimental breast microwave sensing:

1. Develop more robust image quality metrics that describe image contrast, resolution, noise, accuracy, and artifacts. Metrics that utilize distributions of intensities within an image may be more robust than current metrics, which use single-pixel values.
2. Coherence of image quality analysis within the literature should be considered when performing research. Multiple definitions for a common term, or multiple terms having the same definition, only obfuscate the academic literature surrounding breast microwave sensing. We recommend that given their relative prevalence in the literature (as the most commonly used definitions) the following definitions for the SCR, SMR, and localization error, should be used.
  (3) $$\mathrm{SCR}=\frac{T_{\max}}{C_{\max}}$$
  (4) $$\mathrm{SMR}=\frac{T_{\max}}{C_{\mathrm{mean}}}$$
  (5) $$\mathrm{LE}=|r_{\max}-r_{\mathrm{tum}}|$$
3. Compare reconstructed images of tumour-free and tumour-containing phantoms. The use of more robust image quality metrics will assist these comparisons, but even qualitative comparisons should be made.
4. Estimate the specificity of the technique using controlled phantom studies. Despite several patient-based investigations into the sensitivity of the modality, only two estimates of the specificity have been presented. Before patient or volunteer trials are conducted, the specificity must be estimated using controlled phantom studies.
5. Develop objective and robust tumour-detection criteria and utilize these in blind studies to estimate the modality’s diagnostic potential.
6. Published results should be reproducible, and methods should be transparent. Several articles have been missing important information that precludes result reproduction, including information regarding training and evaluation procedures of machine-learning methods, propagation speed estimation methodology (in radar-based image reconstruction), phantom information (dielectric property and geometry information), and reconstruction method. Open-source analysis, as in [53,116,118,119], and open-access datasets, as in [118,119,120], are the best methods for ensuring transparency and reproducibility and should be used when appropriate.
7. Machine-learning applications should be evaluated using diverse datasets and appropriate ML methodologies that are fully described and reproducible. Valid estimates of the diagnostic performance of ML-based diagnosis require adherence to appropriate ML standards, the use of a valid test set, and sufficient dataset diversity. The results from [118,119] should inform future work—the outer tissue geometries were a primary determinant of dataset diversity, and multiple measurements from the same phantom should be constrained to only the training or testing sets, and should not be used in both.

## 7. Conclusions

Breast microwave sensing is a potentially advantageous method for breast cancer detection due to the use of non-ionizing radiation and the relatively large contrast in the dielectric properties of malignant tissues compared to those of healthy tissues. Estimates of the sensitivity of the modality have shown promise in both patient and phantom investigations, but the attention of the research community has been broadly focused on tumour detection rather than diagnostic utility. Existing estimates of the modality’s specificity do not demonstrate significant clinical utility, and range from 20 to 65% [100,120,134]. Specificity is a necessary measure to consider when evaluating the efficacy of a diagnostic tool, particularly in breast cancer screening (where the negative impact of false positives in mammography has been well-documented [1]). Further investigations should quantitatively compare reconstructions of healthy and tumour-containing breasts.

Image quality analysis has been limited to metrics that rely on single-pixel intensity values and/or complete knowledge of the true tissue properties and geometries. These techniques are not suitable for use in blind or double-blind trials to evaluate the efficacy of the modality and are non-robust. Current image quality metrics characterize image contrast (e.g., signal-to-clutter ratio) or accuracy (e.g., localization error). Several aspects of traditional image quality have been neglected, including resolution, noise, and artifacts. The presence of tumour-like artifacts in reconstructed images of healthy breasts is observed in several published studies but has not been adequately addressed.

Machine-learning methods have been explored as potential automated diagnostic tools, but only one article has adhered to fundamental machine-learning methodological standards [120]. Despite fifteen articles reporting estimates of the diagnostic performance (see Table A1(vi)), significant methodological flaws are common. Data contamination, limited dataset diversity, and a lack of an appropriate testing set were observed in the majority of articles exploring machine learning in this review.

Clinical applications of microwave sensing for breast cancer detection are an important and expanding research field attracting international researchers and research group collaborations. Before a microwave-based technique can be considered clinically feasible, the diagnostic utility of the modality must be estimated with respect to sensitivity and specificity. Methods of image quality analysis must extend beyond characterizing contrast, and image artifacts must be analyzed. Despite the promising sensitivity of the modality, much work must be done to characterize image quality and specificity before BMS systems can be considered clinically viable.

## Figures and Tables

**Table 1:** Summary of image quality metrics in breast microwave sensing.

	Signal to Clutter Ratio	Signal to Mean Ratio	Tumour to Fibroglandular Response Ratio	Contrast to Clutter Ratio	Mean-to-Mean Ratio	Localization Error	Mean Squared Error	Structural Similarity Index Measure	Full Width at Half Maximum
**Mathematical Definition ${}^{a}$**	$\frac{T_{\max}}{C_{\max}}$	$\frac{T_{\max}}{C_{\mathrm{mean}}}$	$\frac{T_{\max}^{2}}{F_{\max}^{2}}$	$\frac{T_{\max}^{2}-F_{\max}^{2}}{\sigma }$	$\frac{T_{\mathrm{mean}}}{C_{\mathrm{mean}}}$	$\left|r_{\max}-r_{\mathrm{tum}}\right|$	$\frac{1}{n}\sum_{i}^{n}{\left(I\left(r_{i}\right)-I^{\mathrm{true}}\left(r_{i}\right)\right)}^{2}$	$\frac{\left(2\mu_{1}\mu_{2}+c_{a}\right)\left(2\sigma_{12}+c_{b}\right)}{\left(\mu_{1}^{2}+\mu_{2}^{2}+c_{a}\right)\left(\sigma_{1}^{2}+\sigma_{2}^{2}+c_{b}\right)}$	Various ${}^{b}$
**Publications**	[13,36,61,62,63,65,100,106,116,117,124,139,140,153,165,189,199]	[61,62,63,116,117,124,189]	[26,69,147]	[26,69,147]	[14,99,164,173,178,190]	[14,62,63,65,98,116,117,124,137,139,153,200]	[31,39,72,85,86,89,91,125,180,193]	[176]	[101,114,189,190]
**Measures**	Image contrast	Image contrast	Image contrast	Ratio of contrast to noise	Image contrast	Accuracy of target localization	Image accuracy	Image accuracy	Image accuracy
**Best use**	Contrast maximum target to maximum non-target response	Contrast maximum target to mean non-target response	Contrast maximum target to maximum non-target response	Compare contrast to noise	Compare mean target response to mean non-target response	Describe target positioning error in image	Summary description of image accuracy	Summary description of image accuracy	Describe extent of the highest-intensity response
**Challenges**	Non-robust, requires subjective definition of target region and/or a priori knowledge of tissue geometry.				Requires subjective definition of target region and/or a priori knowledge of tissue geometry.	Requires a priori knowledge of tissue geometry, non-robust.	Requires a priori knowledge of tissue geometries and properties. Only applicable to quantitative reconstruction methods. Only applicable as a summary metric for image accuracy due to summation over the image space.		FWHM may not be limited to a tumour response. The FWHM from images of healthy and tumour-containing breasts may be similar, depending on the geometry of the fibroglandular breast tissues.

^a^ T${}_{\max}$ is the maximum pixel intensity of the tumour response, T${}_{\mathrm{mean}}$ is the mean tumour response, C${}_{\max}$ is the maximum clutter response, C${}_{\mathrm{mean}}$ is the mean clutter response, F${}_{\max}$ is the maximum fibroglandular response, σ is the standard deviation of image intensities, $r_{\max}$ is the position of the maximum image response, $r_{\mathrm{tum}}$ is the known position of the tumour, I(r) is the image intensity at position r, and I${}^{\mathrm{true}}\left(r\right)$ is the true object property at position r. In the definition of the SSIM, $\mu_{i}$ and $\sigma_{i}$ are the average pixel intensity and the standard deviation of the pixel intensities of the image $I_{i}$ and $c_{a},c_{b}$ is defined as $c_{a}={\left(0.01L\right)}^{2}$ and $c_{b}={\left(0.03L\right)}^{2}$, with *L* set to the dynamic range of the intensity values in [176]. ^b^ The FWHM may refer to the volume or area of an image corresponding to the voxels/pixels that have intensities greater than 50% of the maximum image intensity, or it may refer to the FWHM along a particular dimension within the image.

**Table 2:** Estimates of diagnostic performance in image-based tumour detection.

Article	Image Reconstruction Method	Sensitivity Estimate	Specificity Estimate	ROC AUC Estimate	Dataset Information
Poplack et al. [201]	Tomographic	–	–	(80 ± 12)% ${}^{a}$	<130 ${}^{b}$ patients (<80 with abnormal mammography, 50 with normal mammography)
Preece et al. [111]	Modified DAS	74%	–	–	66 patients (all abnormal mammography)
Shere et al. [143]	– ${}^{c}$	76%	–	–	225 patients (all with benign or malignant lesions)
Sani et al. [132]	Huygens Principle	91%	–	–	16 patients (8 healthy breasts, 12 non-healthy breasts)
Sani et al. [133]	Huygens Principle	70%	65% ${}^{d}$	–	45 patients (22 healthy breasts, 29 non-healthy breasts)
Sani et al. [134]	Huygens Principle	74%	62%	–	58 patients (103 breasts, 52 with no radiological findings, 51 with radiological findings)
Sasada et al. [141]	– ${}^{c}$	100%	–	–	5 patients (all with tumours larger than 1 cm)
Adachi et al. [170]	DAS	100%	–	–	9 patients with breast cancer
O’Loughlin et al. [100]	DAS	80%	20%	–	115 phantoms (110 with tumours, 5 without tumours)
Janjic et al. [47]	Qualitative inverse scattering	63%	–	–	115 patients, all with known breast lesions
Reimer et al. [120]	DAS	≤71%	≤44%	–	200 phantom scans (100 healthy; 100 tumour-containing)
	DMAS	≤77%	≤40%	–	
	ORR	≤82%	≤56%	–	
Moloney et al. [94]	TR-MUSIC	87.5%	–	–	24 patients (11 patients with biopsy-proven malignancy, 13 patients with either unaspirated cysts or biopsy-proven benign lesions)

^a^ Reported ROC AUC for tumours with the largest dimension greater than 1 cm. ^b^ Eighty patients with abnormal mammography were scanned with the system, but only the subset of these patients with lesions larger than 1 cm was considered in this analysis. The article does not state the number of patients this included, but the mean and median lesion sizes for the entire sample of patients with abnormal mammography were 11.7 mm and 10 mm, respectively. ^c^ Image reconstruction technique was not stated in the article. ^d^ The authors of [133] reported the FNR (0.35) that corresponded to a particular TPR (0.7). However, the sum of the FNR and TPR must be unity, and this is presumably a textual error within the article.

**Table 3:** Estimates of diagnostic performance in machine learning-based tumour detection.

Article	Classification Algorithm	Sensitivity Estimate	Specificity Estimate	Accuracy Estimate	ROC AUC Estimate	F1 Score	Dataset Information
Santorelli et al. [138]	SVM, LDA	76.71%	67.48%	–	–	–	230 phantom scans
Li et al. [76]	SVM ensemble	97%	99%	–	–	–	150 phantom scans ${}^{a}$
Rana et al. [112]	SVM, MLP, kNN	97.7%	99.7%	98.9%	93.7%	98.6% ${}^{b}$	18 patients (12 healthy breast scans and 11 non-healthy breast scans)
Sani et al. [135]	– ${}^{c}$	88%	59.3%	80.4%	–	86.8% ${}^{b}$	102 breast scans (27 without radiological findings, 75 with radiological findings)
Rana et al. [202]	SVMs with quadratic and Gaussian kernels	97.2%	94.5%	95.5%	–	94.1% ${}^{b}$	61 breast scans (36 with lesion, 25 without lesion) from 34 patients
Dey et al. [188]	PCNN	81.82%	98%	–	–	–	61 breast scans (36 with lesion, 25 without lesion) from 34 patients
Reimer et al. [118]	Logistic regression	(95 ± 6)%	(80 ± 10)%	(85 ± 4)%	(94.4 ± 0.5)%	80.85% ${}^{b}$	249 phantom scans
Al Khatib et al. [53]	CNN	–	–	–	–	92%	1008 phantom scans
Reimer et al. [119]	Logistic regression, CNN, DNN	–	–	–	(90 ± 3)%	–	1008 phantom scans
Martins et al. [82]	kNN, LDA, SVM	–	–	85.00%	–	–	5 phantom scans
Fasoula et al. [196]	QDA	77.1%	100.0%	–	–	–	24 patients (11 patients with biopsy-proven malignancy, 13 patients with either unaspirated cysts or biopsy-proven benign lesions)
Moloney et al. [93]	QDA	87.5%	–	–	–	–	24 patients
Janjic et al. [48]	AdaBoost	79%	77%	78%	74%	78% ${}^{b}$	113 patients (43 with malignant lesions, 70 with benign lesions)
Janjic et al. [49]	Gradient Boosting Ensemble	80%	83%	81%	80%	85% ${}^{b}$	54 patients (25 with malignant lesions, 29 with benign lesions)
Lu et al. [79]	CNN LSTM	–	–	89.5%	–	–	1000 phantom scans

^a^ While 96 healthy patient scans were also performed in [76], synthetic tumour responses were manually introduced into the patient scans. Because the synthetic tumour responses were not experimentally measured, and due to potential differences between true tumour responses and the synthetic tumour responses in [76], only the results from the phantom measurements are reported here. ^b^ Value was calculated to facilitate comparisons with [53] based on the reported sensitivity, specificity, and accuracy within the article, but was not reported explicitly within the article. ^c^ The classifier was not specified in this work. The authors wrote, “An appropriate combination of features...leads to sensitivity of…” [135], but no classification method was specified.

## Data Availability

Not applicable.

## Abbreviations

The following abbreviations are used in this manuscript:

BMS	Breast Microwave Sensing
MRI	Magnetic Resonance Imaging
DAS	Delay-And-Sum
DMAS	Delay-Multiply-And-Sum
IDAS	Improved Delay-And-Sum
SCR	Signal-to-Clutter Ratio
SMR	Signal-to-Mean Ratio
TFRR	Tumour-to-Fibroglandular Response Ratio
CCR	Contrast-to-Clutter Ratio
LE	Localization Error
MSE	Mean Squared Error
FWHM	Full-Width at Half-Maximum
SSIM	Structural Similarity Index Measure
ROI	Region of Interest
ROC AUC	Area Under the Curve of the Receiver Operating Characteristic curve
ORR	Optimization-based Radar Reconstruction algorithm
FNR	False Negative Rate
TPR	True Positive Rate
ML	Machine Learning
SVM	Support Vector Machine
LDA	Linear Discriminant Analysis
QDA	Quadratic Discriminant Analysis
MLP	MultiLayer Perceptron
CNN	Convolutional Neural Network
DNN	Dense Neural Network
kNN	k-Nearest Neighbour
PCA	Principal Component Analysis

## Appendix A

**Table A1:** References for specific claims.

Index	Claim	References
(i)	Papers presenting images without quantitative analysis of image quality	[12,17,27,29,30,32,33,34,35,37,38,40,41,42,43,47,49,50,51,54,55,57,59,64,66,67,73,74,75,77,81,84,87,88,92,95,96,97,102,103,105,107,108,109,110,121,122,123,126,127,128,129,134,136,140,141,144,146,148,150,152,155,156,157,158,160,161,163,166,167,168,169,170,171,172,174,175,177,178,179,180,184,185,187,191,192,194,195,197]
(ii)	Papers presenting image-based analyses with healthy patients or phantoms	[12,25,26,30,33,40,41,42,43,45,49,50,56,63,67,69,72,84,85,87,88,90,91,100,107,108,109,110,120,122,129,131,132,133,134,135,140,142,148,158,162,165,166,169,173,175,184,186,188,190,195,198]
(iii)	Papers that performed quantitative analysis of healthy and unhealthy images	[25,26,45,56,61,63,69,70,72,85,90,91,100,101,120,131,132,133,142,162,165,173,186,190,198]
(iv)	Papers that have reported estimates of the diagnostic performance of image-based BMS	[47,94,100,111,120,132,133,134,141,143,170,201]
(v)	Papers that have estimated image-based diagnostic performance using patient datasets	[47,94,111,132,133,134,141,143,170,201]
(vi)	Papers that have estimated diagnostic performance using machine learning	[48,49,53,76,79,82,93,104,112,118,119,135,138,188,196,202]
(vii)	Papers that have estimated diagnostic performance using machine learning with patient datasets	[48,49,93,112,135,188,196,202]
(viii)	Papers that have estimated diagnostic performance using machine learning with phantom datasets	[53,76,79,82,104,118,119,138]
(ix)	Papers in which data leakage may have occurred	[48,82,135,138,196]
(x)	Papers in which data leakage explicitly occurred	[53,112,113,188,202]
(xi)	Papers that applied ML methods with datasets consisting of multiple measurements of the same phantom	[53,76,79,82,104,118,119,138]
(xii)	Papers that have reported sub-centimeter lesion detection	[13,17,25,26,32,34,36,40,43,45,47,48,51,52,54,57,58,60,64,69,71,83,99,100,114,124,129,148,154,158,163,164,167,168,170,171,172,173,174,175,177,182,187,199]
